# Molecular biology of gliomas: present and future challenges

**Published:** 2014-04-08

**Authors:** R. Altieri, A. Agnoletti, F. Quattrucci, D. Garbossa, F. M. Calamo Specchia, M. Bozzaro, R. Fornaro, C. Mencarani, M. Lanotte, R. Spaziante, A. Ducati

**Affiliations:** 1 Department of Neurosurgery, University of Turin; 2 Department of Medicine, University of Salerno

**Keywords:** genetics, glioma, IDH, metilation, therapy

## Abstract

Malignant brain tumours are one of the most relevant causes of morbidity and mortality across a wide range of individuals. Malignant glioma is the most common intra axial tumor in the adult. Many researches on this theme brought advances in the knowledge of gliomas biology and pathogenesis and to the development of new agents for targeted molecular therapy. Recent studies focused on either tumor metabolism analysis or epigenetic regulation in the pathogenesis or maintenance of brain tumors. This Review summarizes these developments analyzing molecular pathology and possible further developments for targeted therapies.

## I. INTRODUCTION

Astrocyticgliomas are the most common primary brain cancer [1], with an incidence of 3–5 per 100 000 people each year [2]. Gliomas occur in all age groups, but are most prevalent in adults over 45 years of age [3].

Tumors grading is made according to WHO consensus-criteria, in which 4 classes are described, according to malignant behaviour taking into consideration histological features and genetic alterations [4].

- Grade I tumors are biologically benign and total removal leads to recovery;
- Grade II tumors are low-grade malignancies that may follow long clinical courses, but early diffuse infiltration of the surrounding brain renders them often not totally resectable;
- Grade III tumors exhibit aggressive behaviour characterized by increased anaplasia and mithosys over grade II tumors; due to this biological pattern; these tumors have often a quick progression
- Grade IV tumors, also known as glioblastomamultiforme (GBM), exhibit more advanced features of malignancy, including vascular proliferation and necrosis, often refractory to radiotherapy or chemotherapy

On the basis of clinical presentation, GBMs have been further subdivided into primary or secondary GBM subtypes. Primary GBMs account for the great majority of GBM cases in elderly patients, while secondary GBMs are quite rare and tend to occur in younger patients (often below the 45 years old). Primary GBM presents ex novo, with no evidence of prior symptoms or preexistant low grade glioma. Concerning secondary GBMs, the main event is progressive transformation of lower grade astrocytomas in malignant neoplasms. It has been described that up to 70% of grade II gliomas progress to grade III/IV Astrocytomas within 5–10 yr from diagnosis.

Despite different ethiopathogenesis, these two categories, are not distinguishable on hystopathological bases or clinical behaviour.

One of the most relevant hypothesis concerning secondary GBMs, is related to the possibility of successive acquisition of genetic alterations. This hypothesis was proven analyzing selected genetic changes in low grade glioma (LGG), and the presence of the same alterations plus others in in high grade glioma (HGG). Thus the role of specific genetic alteration is supposed to be involved in malignant progression.

Considering grade II, III and IV gliomas, the common behaviour is characterized by invasiveness, thus surgery alone would be not sufficient as curative treatment [5]. The only life-prolonging treatment proven to be effective, consists of the association between radiant therapy and Chemoterapy with the alkilating agent called Temozolamide (TMZ). Despite this, prognosis remains poor with a reported median survival of 14.6 months and a 2-year survival rate of 26.5% [6,7].Tumor recurrence has been observed after 6.9 months (mean) [6], resulting in a median patient survival of just 12–15 months following diagnosis [8]. In order to improve survival, in combination with RT and/or cytotoxic chemotherapy, many targeted drugs were evaluated. In this review, we would like to remark important findings regarding the unique biology of this cancer, considering a neuroscience perspective, focusing on how these insights have influenced latest clinical trials and the search for further treatment options.

## II. METHODOLOGY

A literature search using PubMed MEDLINE database has been performed. The search terms “genetics and glioma”, were combined with the following terms: “IDH, metilation, therapy”.

## III. DISCUSSION

### A. GENETIC ALTERATION IN GLIOMA

#### A.1 Cell cycle dysregulation. RB and P53 pathway

The RB and p53 pathways involved in regulation of the G1-to-S-phase transition, are major targets of inactivating mutations in GBM. The absence of this regulatory system leads to an excessive and inappropriate cell division. This is caused by constitutively active mitogenic signaling effectors, such as phosphoinositide 3′-kinase (PI3K) and mitogen-activated protein kinase (MAPK).

##### A.1.1 The Rb pathway

The Rb1 gene, which maps on chromosome 13q14, is mutated in 25% of HGG and the loss of 13q is characteristic of transition from low-grade to intermediate-grade gliomas [9].

The importance of the inactivation of the RB pathway in glioma progression is evidenced by the near-universal and mutually exclusive alteration of RB pathway effectors and inhibitors in both primary and secondary GBM [10, 11]. However, many in vitro and in vivo assays showed that the neutralization of this pathway alone is insufficient to subvert cell cycle control, thus not sufficient to cause cellular transformation. This is remarkable since other cycle regulation pathways interfere preventing gliomagenesis [12–14].

##### A.1.2 The p53 pathway

The p53 pathway is nearly invariably altered in sporadic (ex novo) GBMs: loss of p53, through either point mutations that prevent DNA binding or loss of chromosome 17p, is a frequent and early event in the pathological progression of secondary GBM [15, 16]. The importance of p53 in gliomagenesis is also demonstrated by the increased incidence of gliomas in Li-Fraumeni syndrome, which is characterized by germline p53 mutations.

Amplification of the p53 antagonists MDM2 and MDM4have also been found in distinct subsets of Tp53 intactGBMs, as well as mutations and/or deletions inthe CDKN2A second locus that encodes p14ARF which is a regulator of p53.

#### A.2 RTKs

The Epidermal Growth Factor and platelet-derived growth factor (PDGF) pathways play an important role in gliomagenesis. Recently targeted therapy against these signalling are under basic and clinical investigation.

##### A.2.1 EGFR

The EGFR is a transmembrane glycoprotein that constitutes one of four members of the ErbB family of tyrosine kinase receptors. EGFR is activated by interaction with ligand to the extracellular domain, leading to receptor dimerization and subsequent activation of tyrosine kinases in the intracellular domain. This results in the stimulation of other downstream signaling effectors including PI3K, Akt, Ras, and mitogen-activated protein kinases (MAPK). All these pathways are involved in cell proliferation, survival, migration, and apoptosis[17].

Approximately 40% of GBMs shows EGFR gene amplification and overexpression [18,19].Amplification of the EGFR gene is often associated with a mutation that encodes for a truncated form of the receptor, known as EGFR variant vIII (EGFRvIII), lacking the extracellular binding domain and leading to constitutive activation of tyrosine kinases [20, 21]. Expression of EGFRvIII correlates with poor survival in GBM patients [22] and promotes glioma cell migration [23], tumor growth, invasion, survival [24] and angiogenesis [25]. Furhtermore, EGFRvIII causes enhanced apoptosis resistance. Activated EGFR is also related to radio-and chemo-resistance in GBM cells [26].

A large number of therapeutic targets have been analyzed in order to block the EGFR signaling pathways, including monoclonal antibodies cetuximab, and TKIs gefitinib, erlotinib and lapatinib. However, the use of EGFR inhibitors, erlotinib and gefitinib, as single agents in patients with progressive GBM has shown no significant survival benefit [27].

##### A.2.2 PDGF receptor (PDGFR)

In addition to the EGFR signaling axis, PDGFRα and its ligands, PDGF-A and PDGF-B, are represented in gliomas, especially in HGG. Concerning expression of PDGFRβ, high levels were reported in proliferating endothelial cells of GBM [28]. PDGF-C and PDGF-D, which require proteolytic cleavage to be activated, are also frequently expressed in glioma cell lines and in GBM tissues [29]. In contrast to the pathway of EGFR; amplification or rearrangement of PDGFRα is a less common event. This justifies that oncogenic deletion mutation of PDGFRα (loss of exons 8 and 9) have been rarely described [30].

Although rare, this mutation has a behaviour similar to EGFRvIII variation: PDGFRα is constitutively active and enhances tumorigenesis. Considering the tumoralcoexpression of PDGF and PDGFR, the main hypothesis considers autocrine and paracrine loops as the main way to favour oncologic transformation. Supportive evidence for a paracrine circuit started by PDGF-B secretion were related to glioma angiogenesis. This has been demonstrated through stimulation of endothelial cells displaying PDGFRβ, in part, to express VEGF [31].On this findings an orally active kinase inhibitor of the 2-phenylaminopyrimidine class such as STI571 (imatinibmesylate) proved to be a potent inhibitor of these oncogenic loops [32, 33]. This drug combined with hydroxyurea in a phase II study, achieved durable anti-tumor activity in some patients with recurrent GBM [34]. Imatinib alone, proved to have minimal activity in malignant glioma [35].

#### A.3 Mitogenicsignaling pathways

Enhanced RTK signalling, whether driven by somatic mutagenesis or other stimuli, seems to play a relevant role in many HGG. These effects are probably mediated through oncogenic PI3K–AKT–mTOR and Ras–MAPK signalling downstream.

This is a remarkable event since it is not infrequent to find molecular components in these downstream networks in HGG [36, 37]. The most common alteration is functional loss of the tumour suppressor gene pTEN, that is the primary negative regulator of PI3K–AKT–mTOR signalling [38, 39].

PTEN is commonly mutated in 20%–40% of GBMs [40]. Thus, targeting the mTOR complex may represent a valuable therapeutic approach for GBM. Actually synthetic analogues of rapamycin (sirolimus), including temsirolimus, everolimus, and ridaforolimus are currently being tested in GBM.

### B. ANGIOGENESIS

GBMs are among the most highly vascular of all solid tumors. Microvascular hyperplasia consists of proliferating endothelial cells that emerge from normal parent microvessels as tufted microaggregates (glomeruloid bodies) accompanied by stromal elements, including pericytes and basal lamina [41]. Microvascular density, a measure of microvascular proliferation, is an independent prognostic factor for adult gliomas [42, 43]. Angiogenesis and its major regulator, VEGF, represents one of the most important therapeutic targets in GBM treatment. Several VEGF/VEGFR inhibitors have been recently developed, including bevacizumab, vatalanib, cediranib, sunitinib, sorafenib, vandetanib, and aflibercept.

Bevacizumab was used in two independent studies as monotherapy for recurrent gliomas. These studies reported favorable 6-month progression-free survival rates of 42.6% [44] and 29% [45], significantly greater than the 18% derived from TMZ use [46]. These studies also showed a reduction in peritumoraledema, allowing a reduction of the corticosteroid dose for symptoms relieve/control [45]. These successes promoted further trials looking at the benefits of adding bevacizumab to radiation and TMZ [47–49]. In literature, there are 114 registered clinical trials using bevacizumab in patients with glioma, typically in combination with other treatment modalities. Unfortunately, the benefit of anti-angiogenic therapies so far remains limited [50]. This may be due to the vascularization supplies of the growing tumor. Moreover, anti-angiogenic therapy showed to produce a more invasive and aggressive tumor cell phenotype, including increased expression of other proangiogenic factors [51], upregulation of pro-invasion proteins such as matrix metalloproteinases[51], activation of proinvasive signaling pathway such as the phosphatidylinositol 3-kinase (PI3K)- and Wnt-signaling pathways [52]. This leads also to an increase in tumor hypoxia by upregulation of hypoxia-inducible factor 1a (HIF1a) [52].

### C. EPIGENETIC: REGULATION OF GENE EXPRESSION THAT ALTERS THE RESPONSE TO TREATMENT

Patients with 6-O-Methylguanine-DNA Methyltransferase (MGMT) promoter methylation who received TMZ and radiation therapy showed a median survival time of 21.7 months compared with 12.7 months for those who had unmethylated MGMT [53]. Two-year survival was 46% with MGMT methylation, compared with 13.8% without MGMT methylation [53].

Increased activity of the MGMT gene inhibits the effects of TMZ, the standard alkylating chemotherapeutic agent used to treat gliomas[53] and explains why some patients fail to benefit from this treatment. In individuals resistant to TMZ treatment, the MGMT promoter [53,54] isunmethylated and transcription of the gene provides sufficient protein to counteract the effects of TMZ. Conversely, patients with a methylated MGMT promoter do not generate the protein and, therefore, are more sensitive to TMZ therapy, particularly if combined with radiation [53]. The recognition that this specific epigenetic modification dramatically alters the potency of a commonly used chemotherapeutic was an important assumption, as it allows a patient-tailored treatment. Unfortunately, the lack of an effective alternative treatment didn’t gave many other options in TMZ refractory patients. It is tempting to speculate that MGMT-mediated DNA repair may itself be considered a potentially valuable therapeutic target for the 50% of patients that express the MGMT gene [55].

### D. INTEGRATED GENOMICS AND SUBSEQUENT ADVANCES

Integrated genomic analysis has also facilitated the identification and characterization of additional genes involved in glioma pathogenesis. Recently, missense mutations in isocitrate dehydrogenase 1 (IDH1) were found in a significant number of GBMs that tend to occur mostly in younger patients with more protracted clinical courses [56]. These point mutations are restricted exclusively to the R132 residue in the active site region of the protein in which they disrupt hydrogen binding with its substrate [56, 57]. Curiously, a separate group of gliomas harbour mutations in the IDH1 homologue IDH2 at the analogous residue (R172). Further investigations have shown that mutations in IDH1 and IDH2 are present in high proportions of grade II and III astrocytic and oligodendroglial tumours (72–100%) along with secondary GBMs (85%), but are largely absent in primary GBMs (5%) [57, 58].

Additionally, IDH mutations are associated with other genomic abnormalities that are typically seen in LGG, such as Tp53 mutation and 1p/19q deletion; they are also mutually exclusive with EGFR amplification and chromosome 10 loss, and multivariate analysis suggests that they are independent favourable prognostic markers [57, 59]. These findings suggest that, although IDH mutations probably contribute to the early evolution of LGG (including those that subsequently progress to higher grade lesions), they seem to have no role in the underlying biology of ex novo GBM. These findings emphatize the pathogenethic differences between these two broad diagnostic categories.

The mechanisms related to mutations in IDH genes and the induction of gliomagenesis are still largely unknown. However, a recent study demonstrated that loss of IDH1 function through point mutation induces hypoxia inducible factor 1α (HIF1α) [60]. This is a component of the hypoxia-responsive transcription factor complex that has been proved to be involved in angiogenesis and tumour growth [61]. By contrast, another recent report has shown that mutant IDH1 proteins exhibit a gain-of-function phenotype by generating R-2-hydroxyglutarate (2HG), a toxic metabolite associated with an increased risk of malignant brain tumours in patients with inherited errors of 2HG metabolism [62].

Although much remains to be analyzed, the identification of IDH mutations in diffuse gliomas, and more recently in acute myeloid leukaemia [63], provided new potential therapeutic targets and emphasized the increasingly compelling link between cancer biology and basic metabolic processes.

### E. GENETIC AND MOLECULAR ALTERATIONS RESULT IN CHANGES IN CELLULAR METABOLISM

It is becoming increasingly evident that at least some of these genetic and molecular alterations result in changes in cellular metabolism. GBMs frequently exhibit increased glucose consumption and lactate production in the presence of oxygen, known as Warburg effect [64] . Activation of PI3K/AKT in GBM cell lines leads to increased glucose uptake and glycolysis [64–66] .

Pyruvate kinase M2 (PKM2), an enzyme that plays a critical role in the glycolytic pathway, is an example of a metabolic enzyme that can affect histone modifications. In EGFR driven glioblastoma, PKM2 translocates to the nucleus and phosphorylates histone 3 at threonine 11 (H3-T11) [67]. This causes dissociation of HDAC3 from the Cyclin D1(CCND1) and c-MYC promoters and subsequent histone acetylation, leading to transcription of CCND1 and c-MYC, and subsequent cell proliferation [67, 68].

More recently, the NADP+ -dependent enzyme IDH1 was found to be mutated in ∼70% of grade II and grade III astrocytomas and oligodendrogliomas, and secondary GBMs [57, 69, 70].

How mutations in IDH1 result in DNA methylation in gliomas is not entirely known. One hypothesis (Fig. 3) is that mutant IDH1 catalyzes the production of 2-hydroxyglutarate (2-HG) from a-ketoglutarate (a-KG). The Jumonji C family of histone lysine demethylases (KDMs) and the TET group of DNA hydroxylases are a-KG-dependent dioxygenases. 2-HG, structurally similar to a-KG [71, 72], is thought to inhibit a-KG-dependent dioxygenase enzymes. Inhibition of these enzymes may result in increased histone methylation marks and DNA methylation contributing to G-CIMP responsible for global changes to DNA methylation, inhibition of histone lysine demethylases, blocks to cellular differentiation, and ultimately, tumorigenesis [73–76].

These observations advocates the possibility that inhibiting IDH mutants might reverse their tumorigenic effects [77] and that the design of effective inhibitors would need to take into account the complex downstream effects of IDH mutations.Recently, to assess this therapeutic possibility in the glioma context, Rohle et al. [78] used AGI-5198, a small molecule inhibitor of the most common IDH mutation in gliomas, IDH1-R132H. Treatment of an oligodendroglioma cell line harboring an endogenous IDH1-R132H mutation with this inhibitor reduced growth in soft agar by 40%–60% and impeded the growth of xenograft tumors derived from that cell line in mice.

Analysis of these tumors showed a reduction in proliferative markers but no change in apoptosis, suggesting that the altered tumor growth was due to failure to proliferate as opposed to cell death. Following treatment, several genes involved in glial differentiation were upregulated and found to have lost repressive histone marks H3K9me3 and H3K27me3 at their promoters, implying that the mutant IDH1 inhibitor is capable of erasing histone modifications that influence gene expression. This study demonstrated that, in this model, targeting mutant IDH1 can impair glioma growth in vivo and this growth inhibition is linked to changes in differentiation.

## IV. CONCLUSION

Gliomas remain one of the most challenging cancers to treat, as demonstrated by the poor improvements in patients survival. This may be partially connected to the fact that current treatment approaches are similar to the ones used for other solid-tumors. This could be counter-productive due to the lack of consideration of the unique biology of gliomas.Despite the strong rationale of clinical trials with genetic targets, most studies have shown very modest results. The negative results reported in the majority of published clinical studies may be explained, at least in part, by single-targeted approaches, which may be somehow limited by such factors as tumor heterogenity and genetic instability. Heterogenity of the GBM cell population, which includes expression of cell surface receptors, as well as proliferative and angiogenic potential, might be attributed to morphological and epigenetic plasticity. On the other hand, there is also evidence for the coexistence of genetically divergent tumor cell clones within tumors. So far, if multiple oncogenic processes are active in distinct tumor subpopulations, single-targeted therapies may provide limited effects. Furthermore, GBM cells may exhibit significant genetic instability, possibly leading to resistance of single-targeted agents by switching to alternative molecular pathway. Recently, however, a new paradigm is emerging in cancer biology, represented by convergence of multiple metabolic pathways and epigenetic regulation. The complex connection between the metabolic state of a cancerous cell and its epigenetic machinery represents a novel mechanism, by which the normal control of cell proliferation/differentiation can be disrupted. Discovering the dynamics and intricacies of these processes may help us in developing a better understanding of gliomas, thus permitting the knowledge of novel therapeutic targets to effectively fight these highly aggressive tumors.

We suggest that new trends on HGG targeted therapies should not focus on a single genetic pathway, but should try to interact in different molecular pathways.We think that HGG biology has many shortcuts and secret passages, not yet fully discovered.

## Figures and Tables

**TABLE I:** GENETIC ALTERATION IN GLIOMA

*Cell cycle dysregulation*	*RTKs*	*Mitogenicsignaling pathways*
***Rb1***➔ mutated in 25% of HGG; loss of 13q ➔ transition from LGG to intermed iate- grade gliomas	***EGFR*** gene amplification and overexpression➔ 40 % of GBMs	***PTEN***mutation ➔ 20% 40% of GBMs
***p53***➔ne arly invariabl y altered in ex novo GBMs; loss of p53➔ early event in progress ion of secondar y GBM	***PDGF*** and ***PDGFR***coexpression ➔autocrine and paracrine loops determine oncologic transformation. ***PDGF-B***➔ glioma angiogenesis	***PI3K***–***AKT***–***mTOR***signallingco stitutively activated by stimulation of RKTs and unsuppressed by PTEN

**TABLE II:** IDH1 and IDH2 MUTATION

AstrociticangOligodendroglial tumours (WHO II & III)	72–100%
secondary GBMs	85%
primary GBMs	5%

**TABLE III:** IDH1 MUTATION

*Loss of function*	*Gain of function*
HIF1α➔angiogenesis and tumour growth	2HG➔increased histone methylation➔blocks to cellular differentiation, andtumorigenesis

**TABLE IV:** BIOLOGICAL TARGET THERAPY

*RTKs*	*PTEN pathway*	*Angiogenesis*	*IDH1*
Erlotinib, Gefinitib➔ EGFR; Imatinib+Hyd roxyurea➔PDGF	Temsirolimus, everolimus, ridaforolimus➔mTOR	Bevacizum av➔VEGF	AGI-5198➔ID H1-R132 H
